# Type II Grass Carp Reovirus Rapidly Invades Grass Carp (*Ctenopharyngodon idella*) via Nostril–Olfactory System–Brain Axis, Gill, and Skin on Head

**DOI:** 10.3390/v15071614

**Published:** 2023-07-23

**Authors:** Wentao Zhu, Meihua Qiao, Meidi Hu, Xingchen Huo, Yongan Zhang, Jianguo Su

**Affiliations:** 1 College of Fisheries, Hubei Hongshan Laboratory, Huazhong Agricultural University, Wuhan 430070, China; zhuwentao@webmail.hzau.edu.cn (W.Z.); qiaomeihua369@163.com (M.Q.); humeidi@webmail.hzau.edu.cn (M.H.); huoxingchen2020@webmail.hzau.edu.cn (X.H.);; 2 Laboratory for Marine Biology and Biotechnology, Pilot National Laboratory for Marine Science and Technology, Qingdao 266237, China

**Keywords:** *Ctenopharyngodon idella*, grass carp reovirus, invasion portal, nostril, gill

## Abstract

Type II grass carp reovirus (GCRV-II) with high pathogenicity and infectivity causes severe hemorrhagic disease, which leads to extensive death in the grass carp and black carp aquaculture. However, the early invasion portal remains unclear. In this study, we explored the invasion portal, time, and pathway of GCRV-II by immersion infection in grass carp. Through the detection of the infected grass carp external body surface tissues, most of them could be detected to carry GCRV-II within 45 min except for the skin covered by scales. Further shortening the duration of infection, we proved that GCRV-II rapidly invades through the nostril (especially), gill, and skin on head at only 5 min post-immersion, rather than merely by adhesion. Subsequently, visual localization investigations of GCRV-II were conducted on the nostril, olfactory system (olfactory bulb and olfactory tract), and brain via immunofluorescence microscopy and transmission electron microscopy. We found that few viruses were located in the nostril at 5 min post-immersion infection, while a significantly increased quantity of viruses were distributed in all of the examined tissues at 45 min. Furthermore, the semi-qRT-PCR and Western blotting results of different infection times confirmed that GCRV-II invades grass carp via the nostril–olfactory system–brain axis and then viral replication unfolds. These results revealed the infection mechanism of GCRV-II in terms of the invasion portal, time, and pathway in grass carp. This study aims to understand the invasion mode of GCRV-II in grass carp, thus providing theoretical support for the prevention and control strategies of hemorrhagic disease.

## 1. Introduction

Grass carp reovirus (GCRV) is a member of the Reoviridae family, which can cause severe hemorrhagic disease in grass carp (*Ctenopharyngodon idella*), black carp (*Mylopharyngodon piceus*), rare minnow (*Gobiocypris rarus*), etc. GCRV is comprised of 11 double-stranded RNA genome segments surrounded by multiple concentric proteins: three large (S1–S3), three medium (S4–S6), and five small segments (S7–S11). These genome segments encode both structural proteins and non-structural proteins of the virus [1,2]. GCRV can be divided into three types: type I, II, and III [3]. Among them, type II GCRV (GCRV-II) is the most widely distributed and the most virulent strain. VP4 is the major outer capsid protein encoded by the GCRV-II segment 6 gene, and it is one of the signature proteins used to detect the presence of GCRV [4,5]. The clear understanding of the target organs and infection routes of virus invasion can effectively prevent and control the outbreak of virus-induced diseases [6]. Previous study has reported that GCRV-II can infect most tissues of grass carp, mainly affecting the brain (especially), heart, and eyes of grass carp, and establishing latent infection in the brain tissue [7]. However, the fundamental problem of identifying the invasion portal and pathway of GCRV-II invasion in grass carp remains unknown.

Viral infections of the central nervous system (CNS) are rare and often devastating, leading to death or permanent neurologic damage. Neurotropic viruses may gain access to the CNS via several routes including anterograde neuronal spread through sensory nerves [8], across the blood–brain barrier as free virions, or via the entry of infected immune cells [9,10]. In mammals, studies on the kinetics of neurotropic viral invasion have identified the olfactory bulb as the earliest site of CNS infection. Indeed, the most direct conduit from the periphery to the brain occurs at the level of the olfactory neuroepithelium within the nasal cavity, where cell bodies of olfactory sensory neurons reside and send their axons into the CNS to synapse with dendrites of mitral neurons within the olfactory bulb. This route of entry was first investigated in the early 1900s in the context of poliovirus infection. Since the first study demonstrated that the virus establishes its initial focus in the olfactory bulb [11], it has subsequently been reported that instillation of poliovirus into the nasal cavity, but not the stomach, leads to manifestations of CNS disease [12]. Evidence from a variety of animal models and human cases has indicated that many DNA and RNA viruses, including herpesviruses [13], vesicular stomatitis and rabies viruses [14], paramyxoviruses parainfluenza and measles viruses [15], Venezuelan equine encephalitis and chikungunya viruses [16], La Crosse virus [17], and influenza A [18], are initially detected within the olfactory bulb during neuroinvasive infection. These studies suggest that the nostril and olfactory system may amount to a key target invasion portal in viral infections.

The olfactory system of teleost fish is a paired organ consisting of two olfactory bulbs that sit inside the nasal cavity and are connected to the CNS via the olfactory tract [19,20]. The olfactory systems of different teleost species show considerable morphological diversity but the general anatomical and molecular organization of olfactory system appears well conserved across fish taxa [21]. Since water constantly circulates through the nasal cavities to detect odorants, the fish olfactory organ is constantly exposed to any microorganisms present in the water. Although the materials entering the nostril cannot directly enter the internal environment of the fish body, as the most important external surface tissue, the nostril is still at great risk of infection by external pathogens. Therefore, the nostril and olfactory system can amount to an important route for pathogen entry into fish. However, whether GCRV-II invasion can begin in the nostril and unfold along the olfactory system remains unknown.

This study investigated the invasion portal, time, and pathway of GCRV-II in grass carp. We simulated natural infection conditions to establish a GCRV-II-infected immersion model, and we found that GCRV-II can invade the main external surface tissues of grass carp nostril, gill, eye, mouth, skin on head, and cloacal pore, except skin covered by scales and skin under lateral line scales within 45 min. Further shortening the infection time to pinpoint the invasion portal, we found that only 5 min after infection, GCRV-II invades the nostril (especially), gill, and skin on head in grass carp, and this invasion is distinct from adherence to tissue surfaces. To further investigate the invasion pathway of GCRV-II, we studied the invasion of the olfactory system (olfactory bulb and olfactory tract) by GCRV-II. By locating and tracking GCRV-II in grass carp within 45 min, we found that GCRV-II can gradually invade grass carp along the nostril–olfactory system–brain path. Moreover, the GCRV-II invasion of this axis will gradually increase after six hours. These results reveal for the first time the invasion portal and invasion pathway of GCRV-II infecting grass carp, which provides insights into the invasion mechanism of the aquatic pathogen in fish.

## 2. Materials and methods

### 2.1. Fish and Sampling

Healthy grass carp and common carp (*Cyprinus carpio*, control) individuals (weighing 25–30 g; Hanchuan City, China) were purchased and kept temporarily. The fish displayed no symptoms of disease or physical damage on their body surface. All fish used in this study were cultured and cared for at HZAU in accordance with Institutional Animal Care and Use Committee guidelines that were approved by the Scientific Ethic Committee of Huazhong Agricultural University.

Before the formal experiment, they were temporarily raised in a recirculating freshwater system at 28 °C for more than two weeks and fed twice a day with a commercial pellet diet at a rate of 2% body weight. In the formal experiment, the outer body surface tissues of grass carp, including gill, eye, mouth, nostril, skin on head, skin under lateral line scales, skin covered by scales, and cloacal pore, were collected and immediately (at 1 h post infection) washed with sterile PBS 3 times, and used for GCRV-II detection. For further investigation, the nostril (only the nostril tissue cavity, about 0.5 cm), olfactory bulb (contains no excess tissue), olfactory tract (contains no excess tissue), brain, and gill tissues were collected (at different time post infection, including 15 min, 25 min, 35 min, 45 min, 6 h, 12 h, 24 h, and 48 h) and washed for GCRV-II detection. Four fish were sampled for testing, and the test results of one of them were taken for display.

For the following semi-quantitative RT-PCR (semi-qRT-PCR) or quantitative RT-PCR (qRT-PCR) experiments, the samples were soaked in TRIzol and placed in a –80 °C freezer for RNA extraction. For the Western blotting (WB) experiments, the samples were prepared as tissue protein samples and placed at –20 °C. Uninfected fish nostril tissue was used as the control in the PCR and WB assay. For the transmission electron microscopy (TEM) observation, the samples were placed in 10% neutral formaldehyde solution fixed at 4 °C overnight, and the last was fixed with glutaraldehyde solution for experiment.

### 2.2. Virus and Immersion Infection

GCRV-097 (GCRV-II) was used in this study [22]. In order to simulate the pattern of natural infection, we carried out immersion infection with grass carp. Briefly, 3 L of water (control) or GCRV-II solution (1 × 10^6^ TCID50/mL, 4 μL/g) was poured into a 5 L beaker at 28 °C. The grass carp was soaked for infection, time was strictly controlled, and the grass carp was taken out in time for sampling.

### 2.3. Semi-qRT-PCR and qRT-PCR

Total RNAs were extracted with TRIzol (Simgen) and converted into cDNA using the Reverse Transcription Kit HiScript II Q RT SuperMix + gDNA wiper (Vazyme Biotech Co., Ltd., Nanjing, China) and hexamer primer. Semi-qRT-PCR was performed by a 20 μL reaction system, including 10 μL of Mix (TSINGKE), 8 μL of nuclease-free water, 1 μL of diluted cDNA (200 ng), and 0.5 μL of each gene-specific primer (10 μM). PCR reaction conditions were as follows: 94 °C, 5 min; 94 °C for 30 s, 58 °C for 45 s, and 68 °C for 2 min, for 35 cycles; then extended at 72 °C for 10 min. Finally, PCR products were subjected to gel electrophoresis and photographed. β-actin was used as the internal gene. qRT-PCR was established in a Roche LightCycler^®^ 480 system, and 18S rRNA was employed as an internal control gene for cDNA normalization [23]. All cDNA concentrations were adjusted to 50 ng/μL.

qRT-PCR amplification was carried out in a total volume of 15 μL, containing 7.5 μL of BioEasy Master Mix (SYBR Green) (Hangzhou Bioer Technology Co., Ltd. Hangzhou, China), 3.1 μL of nuclease-free water, 4 μL of diluted cDNA (200 ng), and 0.2 μL of each gene-specific primer (10 μM). mRNA expression levels were normalized to the 18S rRNA expression level, and data were analyzed using the 2^−ΔΔCT^ method. Semi-qRT-PCR and qRT-PCR primers were designed by Primer Premier 5 software based on GenBank gene sequence information, as shown in Table 1.

### 2.4. WB Analysis

The anti-VP4 mouse polyclonal antibody (Ab) was prepared and conserved in our lab [7]. Anti-β-tubulin rabbit monoclonal Ab was purchased from Abcam (Cambridge, UK). For WB analysis, protein extracts were separated by 8% SDS-PAGE gels and transferred onto nitrocellulose membranes (Millipore). Membranes were blocked in fresh 2% bovine serum albumin dissolved in a TBST buffer (25 mM Tris-HCl, 150 mM NaCl, 0.1% Tween 20 [pH 7.5]) at 4 °C overnight, then incubated with the appropriate indicated primary Ab for 2 h at 37 °C. Nitrocellulose membranes were washed three times with a TBST buffer and incubated with a secondary Ab for 1 h at 37 °C. After washing four times with a TBST buffer, nitrocellulose membranes were scanned and imaged by Image Quant (GE, America). Results were obtained from five independent experiments.

### 2.5. Immunofluorescence Microscopy

The nostril, olfactory bulb, olfactory tract, and brain tissues of immersion infected grass carp were sectioned and fixed with xylene for 15 min, soaked with ethanol for another 15 min, then washed three times with phosphate-buffered saline (PBS). Next, sections were denatured with 0.01 M SSC (1× SSC is 0.15 M NaCl plus 0.015 M sodium citrate) at 95 °C for 15 min. Then, sections were incubated with 5% bovine serum albumin at 37 °C for 1 h. After incubation, sections were incubated with primary mouse anti-VP4 antibodies (1:1000) and secondary antibody fluorescein isothiocyanate (FITC)-conjugated goat anti-mouse IgG (1:200; ABclonal) at 37 °C for 1 h, respectively, and stained with 1 mg/mL 4′,6-diamidino-2-phenylindole (DAPI) (1:1000) at 37 °C for 10 min. After washing three times with PBS, sections were observed through the UltraVIEW VoX 3D Live Cell Imaging System (PerkinElmer).

### 2.6. Transmission Electron Microscopy

The nostril, olfactory bulb, olfactory tract, and brain tissues of immersion infected grass carp were fixed with 2.5% glutaraldehyde in a 0.1 M phosphate buffer (pH 7.2) at 4 °C for 24 h to observe the virus particles. Ultrathin sections were prepared as previously described [24]. Images were viewed on an HT-7700 TEM (Hitachi, Japan).

### 2.7. Statistical Analysis

Statistical analyses and presentation graphics were crafted using the GraphPad Prism 8.0 software (GraphPad Software). Results are presented as mean ± standard deviation (SD) for at least three independent experiments.

## 3. Results

### 3.1. Immersion Infection of GCRV-II Can Lead to Rapid Invasion in the External Body Surface Tissues of Grass Carp

**Figure 1:**
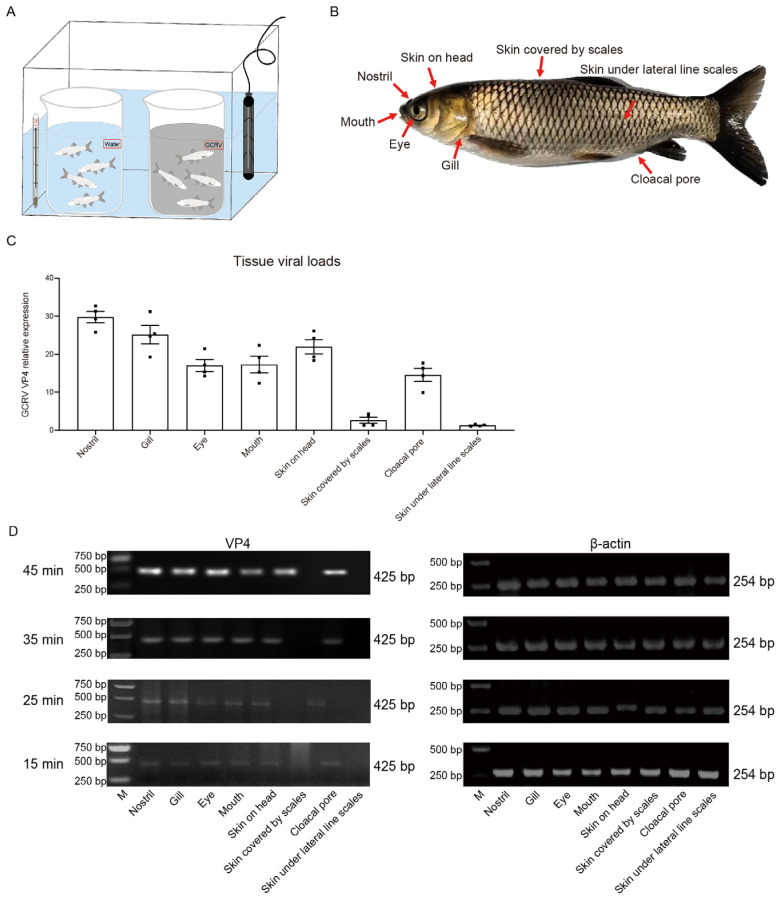
GCRV-II can invade the external body surface tissues in grass carp within 45 min. (**A**) Experiment device for the immersion infection. (**B**) Schematic diagram of external body surface tissues of grass carp for sampling, including gill, eye, mouth, nostril, skin on head, skin under lateral line scales, skin covered by scales, and cloacal pore. (**C**) Tissue viral loads of GCRV-II in the external body surface tissues at 45 min post immersion infection with GCRV-II via qRT-PCR. VP4 was employed as the marker gene of GCRV-II, and 18S rRNA was used as an internal gene (*n* = 4). (**D**) mRNA expression levels of VP4 in the external body surface tissues at 45, 35, 25, 15 min post immersion infection with GCRV-II via semi-qRT-PCR. β-actin was employed as the internal gene. The experiments were repeated in triplicate.

To investigate the invasion portal of GCRV-II to grass carp, we immersed grass carp in GCRV-II or ordinary water at 28 °C to simulate the natural infection condition. This small device can strictly control the consistency of each infection experiment, and it is easy to control the infection time (Figure 1A). Eight immersion infected external body surface tissues, including nostril, gill, eye, mouth, skin on head, skin covered by scales, skin under lateral line scales, and cloacal pore, which were in direct contact with GCRV-II, were sampled for detection of GCRV-II infection (Figure 1B). Tissue viral loads after GCRV-II infection for 45 min were detected via qRT-PCR. The results showed that GCRV-II exists in most examined tissues except for skin covered by scales and skin under lateral line scales, with the highest relative viral loads in the nostril (Figure 1C). The infection time of immersion was further shortened from 45 min to 15 min. We found a gradual increase in GCRV-II in these tissues via semi-qRT-PCR detection (Figure 1D). These results indicated that GCRV-II can invade the external body surface tissues of grass carp within 45 min and gradually establish infection.

### 3.2. Nostril as the Major Invasion Portal of GCRV-II Can Be Invaded in 5 min

**Figure 2:**
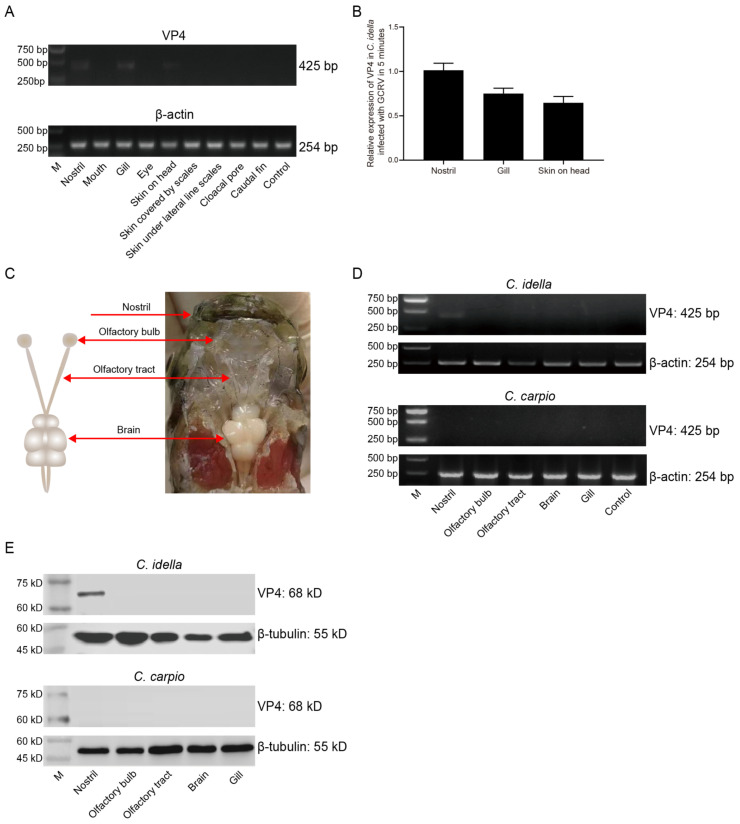
GCRV-II invades the nostril of grass carp at 5 min. (**A**) mRNA expression levels of VP4 in the external body surface tissues at 5 min post immersion infection with GCRV-II via semi-qRT-PCR. Uninfected grass carp nostril tissue was used as the control. β-actin was employed as the internal gene. (**B**) Relative mRNA expression levels of VP4 in nostril, gill, and skin on head, analyzed via ImageJ (*n* = 4). (**C**) Schematic diagram of nostril–olfactory system (olfactory bulb and olfactory tract)–brain in grass carp. (**D**,**E**) Relative mRNA (**D**) and protein (**E**) expression levels of VP4 in the nostril, olfactory system, brain, and gill at 5 min post immersion infection for grass carp and common carp via semi-qRT-PCR and WB. Uninfected grass carp or common carp nostril tissue was used as the control. VP4 was employed as a marker gene for GCRV-II. β-actin and β-tubulin were used as the internal gene, respectively. The experiments were repeated in triplicate.

In order to further explore the main invasion portal of GCRV-II accurately, we reduced the infection time to 5 min. The invasion of grass carp by GCRV-II was detected via semi-qRT-PCR, and the results showed that the presence of the virus can only be detected in the nostril, gill, and skin on head (Figure 2A). Via ImageJ analysis, the highest viral load was observed in the nostril tissue at 5 min post GCRV-II immersion infection, followed by gill and skin on head (Figure 2B), indicating that the nostril may be the main invasion portal of GCRV-II in grass carp. Fish nostrils are connected to brain tissue via the olfactory system. Therefore, we sampled the nostril, olfactory system (including olfactory bulb and olfactory tract), and brain of grass carp to investigate the invasion pathway of GCRV-II infection (Figure 2C). We detected the invasion of grass carp by GCRV-II via semi-qRT-PCR and took the same tissues of common carp (*Cyprinus carpio*) (GCRV-II cannot infect) as the control to avoid the situation wherein GCRV-II only adheres to the tissue surface. The results showed that GCRV-II can only be detected in the nostril tissue of grass carp after immersion for 5 min, not in the tissues of common carp (Figure 2D). Similarly, the detection of VP4 protein of GCRV-II by WB also showed consistent results (Figure 2E). These results indicated that the nostril is the main invasion portal of GCRV-II infection in grass carp, and GCRV-II invasion can occur at 5 min post viral immersion.

### 3.3. GCRV-II Is Located in Nostril, Olfactory System, and Brain within 45 min

**Figure 3:**
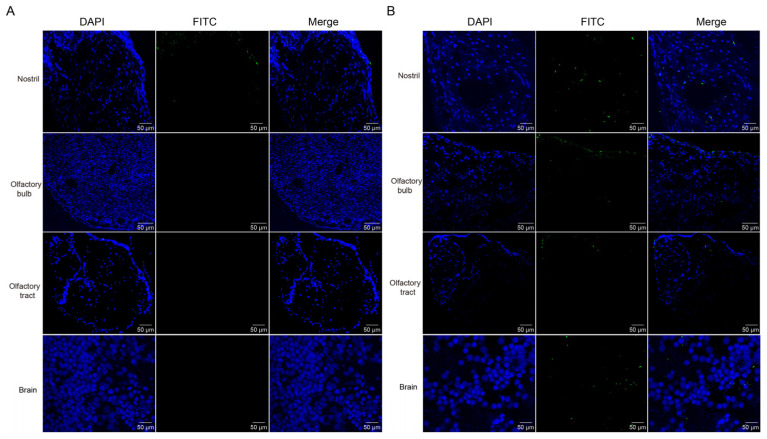
Immunofluorescence detection of GCRV-II in the nostril-olfactory system-brain axis. (**A**,**B**) Immunofluorescence detection of VP4 in the nostril, olfactory bulb, olfactory tract, and brain tissues at 5 min (**A**) and 45 min (**B**) post immersion infection in grass carp. Blue represents the DAPI-stained nucleus, and green stands for the FITC-labeled fluorescent secondary Ab to detect VP4 proteins of GCRV-II (scale bar: 50 μm).

**Figure 4:**
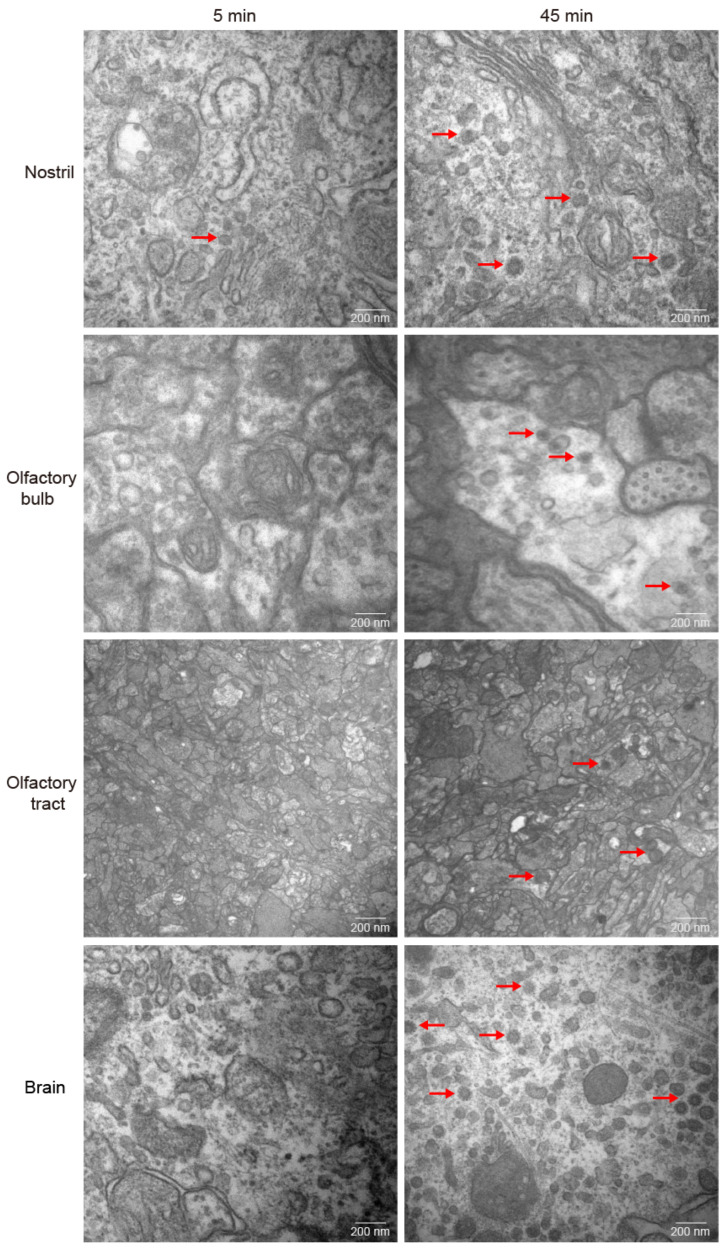
Distribution of GCRV-II in the nostril–olfactory system–brain axis via TEM. Micrographs of grass carp nostril, olfactory bulb, olfactory tract, and brain tissues at 5 min (**left**) and 45 min (**right**) post immersion infection with GCRV-II via TEM (scale bar: 200 nm). The red arrows show the virions of GCRV-II.

Previous study has discovered that GCRV-II is abundant in the brain post infection [7]. GCRV-II preferentially enters the body through the nostril tissue on the external body surface of grass carp in 5 min. Then, we wondered whether it can further disseminate via nostril, olfactory system, and brain tissues. First, we examined GCRV-II in nostril, olfactory bulb, olfactory tract, and brain tissues at 5 min and 45 min post immersion infection by immunofluorescence. The results showed that only a small amount of GCRV-II was found to be located in nostril tissue, and almost no green fluorescence was observed in other tissues at 5 min post immersion infection (Figure 3A). At 45 min post immersion, more of the virus was found in nostril, olfactory bulb, olfactory tract, and brain tissues (Figure 3B). Further, we investigated the GCRV-II distribution via TEM. Virions were only found in the nostril at 5 min after infection, while the presence of virions was obviously found in nostril, olfactory bulb, olfactory tract, and brain tissues at 45 min after infection (Figure 4). These results suggest that GCRV-II can locate in the nostril, olfactory system, and brain within 45 min after entering through the nostril, and they inspired us to further investigate GCRV invasion along this axis.

### 3.4. GCRV-II Invades Brain along the Nostril–Olfactory System Path within 45 min

**Figure 5:**
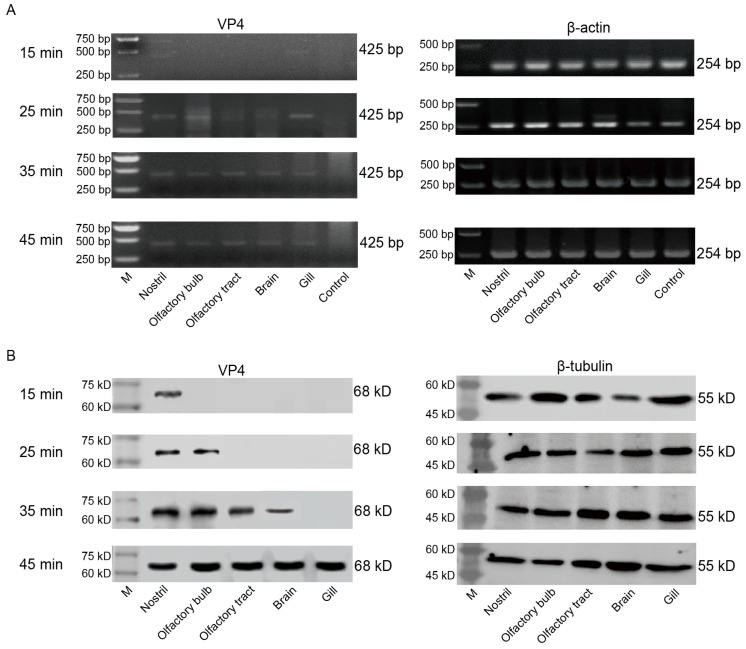
GCRV-II invades the brain along the nostril–olfactory system–brain path within 45 min. (**A**,**B**) Relative mRNA (**A**) and protein (**B**) expression levels of VP4 in the nostril, olfactory system, brain, and gill tissues at 15, 25, 35, and 45 min post immersion infection in grass carp by semi-qRT-PCR and WB. Uninfected grass carp nostril tissue was used as the control. VP4 was employed as a marker gene for GCRV-II. β-actin and β-tubulin were used as the internal genes, respectively.

GCRV-II was localized in the nostril, olfactory bulb, olfactory tract, and brain within 45 min post infection, and we wanted to further confirm whether it invades and infects brain tissue along this axis. We sampled grass carp tissues after immersion infection for 15, 25, 35, and 45 min, and detected tissue viral loads via semi-qRT-PCR and WB. Semi-qRT-PCR results showed that GCRV-II was detectable in all these tissues at 25 min and increased over time (Figure 5A). Similarly, WB results showed that the virus was only detectable in the nostril at 15 min post infection, it was then gradually detectable along the nostril–olfactory bulb–olfactory tract–brain route and in the gill within 45 min (Figure 5B). These results indicated that GCRV-II invades grass carp along the nostril–olfactory system–brain axis and the gill within 45 min post immersion infection.

### 3.5. The Infection of GCRV-II in the Nostril–Olfactory System–Brain Was Aggravated after 6 h

**Figure 6:**
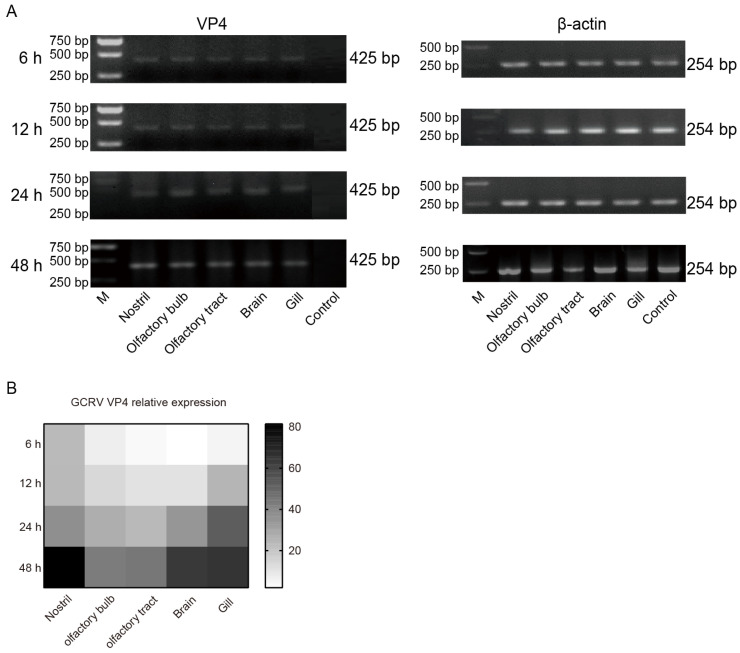
The infections of GCRV-II in the nostril–olfactory system–brain were aggravated after 6 h. (**A**) Relative mRNA expression levels of VP4 in the nostril, olfactory system, brain, and gill tissues at 6, 12, 24, and 48 h post immersion infection in grass carp via semi-qRT-PCR. Uninfected grass carp nostril tissue was used as the control. β-actin was employed as the internal gene. (**B**) Tissue viral loads of GCRV-II in the nostril, olfactory system, brain, and gill tissues at 6, 12, 24, and 48 h post immersion infection in grass carp via qRT-PCR. VP4 was employed as the marker gene of GCRV-II, and 18S rRNA was used as an internal gene (*n* = 4).

In order to further investigate the invasion caused by GCRV-II, grass carp tissue samples were taken at 6, 12, 24, and 48 h post immersion infection. mRNA RNA levels of the virus were detected via semi-qRT-PCR. The results showed that the invasion and replication of GCRV-II in these tissues increased significantly from 6 h to 48 h post infection (Figure 6A). Further, we examined the tissue viral loads after infection via qRT-PCR, and the results also showed that the presence of GCRV-II was significantly increased in nostril, olfactory bulb, olfactory tract, brain, and gill tissue over time (Figure 6B). These results suggested that GCRV-II invades through the nostril–olfactory system–brain axis, and gill, and viral replication gradually occurs in these tissues.

## 4. Discussion

Clarifying the invasion portal, time, and pathway provides crucial theoretical support for studying the mechanisms of disease outbreak. In order to investigate this fundamental question of GCRV-II invasion in grass carp, we simulated natural infection condition by immersion infection. Eight external surface tissues (direct exposure to water environment with microbes) were sampled to determine the invasion portal of GCRV-II. At 45 min post infection, the presence of GCRV-II was found in most of the tested tissues, except for areas covered by scales. By continuously shortening the infection time, we finally found that GCRV-II existed in the nostril, gill, and skin on head at 5 min, and by further WB analysis, we proved that GCRV-II establishes rapid invasion in the nostril, which was different from adhesion. Therefore, we identified the nostril as the early and main invasion portal of GCRV-II infection.

That the nostril acts as pathogen invasion portal is not uncommon in mammals, see for examples: poliovirus, herpesvirus, and several types of rhabdovirus, etc. [25]. The nostril is also one of the main organs of pathogen invasion in teleost fish. The teleost olfactory organ can be infected by bacteria, parasites and viruses [26] as evidenced from natural infections or experimental challenges. For example, *Edwardsiella ictaluri* can infect channel catfish (*Ictalurus punctatus*) via the olfactory route, producing olfactory sacculitis upon acute immersion exposure [27]. *Streptococcus iniae* can enter and affect the nares in hybrid striped bass (*Morone chrysops* × *Morone saxatilis*) [28,29] and tilapia (*Oreochromis niloticus*) [30], in addition, *Renibacterium salmoninarum* can also cause lesions in the nares of salmonid fish. Several viral pathogens have been isolated from the olfactory organ of fish, including the following RNA viruses: infectious hematopoietic necrosis virus (IHNV) [31,32], viral hemorrhagic septicemia virus (VHSV) [33], and grouper nervous necrosis virus [34]. In addition, a DNA virus, the sturgeon iridovirus, has also been reported to infect the olfactory organ of fish [35]. With regards to parasites, natural infections of the ciliated protozoan *Uronema nigricans* have been reported in the tuna (*Thunnus maccoyii*) olfactory organ [36]. In the case of the GCRV-II infection in this study, the rapid invasion at 5 min results in the highest viral tissue load in the nostril, indicating the pertinence of the viral invasion to the target organ.

The olfactory system of teleost fish is a paired organ consisting of two olfactory bulbs that sit inside the nostrils, and olfactory bulbs are connected to the CNS via the olfactory tracts [19,20]. Previous study revealed that GCRV-II establishes latent infection in the brain of grass carp, and the brain is the main target organ for GCRV-II infection [7]. Fish nostrils are connected to the brain via the olfactory system, as a consequence, viruses have the chance to invade brain tissue by nostril-olfactory system-brain route and cause subsequent infection. In the current study, by tracking the locations at different time points, GCRV-II was observed to invade along the nostril–olfactory system (olfactory bulb and olfactory tract)–brain axis within 45 min.

In the investigation, skin on head and gill tissue also showed a certain viral load 5 min after GCRV-II infection. Skin on head is not covered by fish scales compared to other uninfected tissue, thus increasing the risk of infection, as GCRV-II may have the ability to invade skin tissue that is not protected by scales. Gill tissue also plays a major role in GCRV-II invasion compared with other tissues. Gills are exposed to the outside environment and rapidly exchange gas in water. We hypothesized that GCRV-II infects gill and other exposed skin, enters the blood, and subsequently disseminates through blood circulation system (plasma and blood cells). GCRV-II easily breaks through the thin monolayer flattened epithelial cells in branchial lamella and enters blood circulation, which is fast in gills, thus, the virus is transported to other tissues; hence, the virus can be detectable at 5 min via semi-qRT-PCR but detectable at 45 min via WB. Previous study has indicated that GCRV-II can infect leukocytes [24]. Thus, GCRV-II can pass the blood–brain barrier. GCRV-II entering blood can circulate throughout the whole body.

## 5. Conclusions

GCRV-II can invade the exposed external surface (without scale cover) in grass carp within 45 min under the condition of immersion which simulates natural infection. GCRV-II rapidly invades the nostril (especially), gill, and skin on head in grass carp within 5 min, which is distinguished from virus adhesion. GCRV-II was observed to invade along the nostril–olfactory system (olfactory bulb and olfactory tract)–brain axis within 45 min. The gills are also an important portal for GCRV-II invasion, which is then disseminated via the blood circulation system. In addition, GCRV-II obviously accumulates in the invasion portals after 6 h. This study provides insights into the mechanisms of GCRV-II invasion and contributes to the prevention and control strategies of GCRV pandemics.

## Figures and Tables

**Table 1:** Primers used in this study.

Gene Name	Sequence (5′ to 3′)	Accession Number	Application
VP4	F: GATGGCGATAAAGGG	OM854797.1	Semi-qRT-PCR
	R: CGCTGGGTTGATAGGACA		
β-actin	F: TAACCCTCGTAGATGGGCACAGT	M25013	
	R: ATCTGGCATCACACCTTCTACAAC		
VP4	F: CGAAAACCTACCAGTGGATAATG	OM854797.1	qRT-PCR
	R: CCAGCTAATACGCCAACGAC		
18S rRNA	F: ATTTCCGACACGGAGAGG	EU047719	
	R: CATGGGTTTAGGATACGCTC		

## Data Availability

Not applicable.
